# Patient-reported outcomes from the phase II FAST trial of zolbetuximab plus EOX compared to EOX alone as first-line treatment of patients with metastatic CLDN18.2+ gastroesophageal adenocarcinoma

**DOI:** 10.1007/s10120-020-01153-6

**Published:** 2021-03-23

**Authors:** Florian Lordick, Salah-Eddin Al-Batran, Arijit Ganguli, Robert Morlock, Ugur Sahin, Özlem Türeci

**Affiliations:** 1 University of Leipzig Medical Center, University Cancer Center (UCCL), Leipzig, Germany; 2 Institut für Klinische Krebsforschung IKF Am Krankenhaus Nordwest, Frankfurt, Germany; 3 Astellas Pharma, Inc, Northbrook, IL USA; 4 YourCareChoice, Ann Arbor, MI USA; 5 TRON – Translational Oncology at the University Medical Center of the Johannes Gutenberg University Mainz, Mainz, Germany; 6 University Medical Center of the Johannes Gutenberg University Mainz, Mainz, Germany; 7 Biopharmaceutical New Technologies (BioNTech) Corporation, Mainz, Germany; 8 CI3 – Cluster of Individualized Immune Intervention; formerly of Ganymed Pharmaceuticals GmbH, Mainz, Germany

**Keywords:** Stomach cancer, Quality of life, Biomarkers, Antibodies, Monoclonal, Patient-reported outcomes

## Abstract

**Background:**

Zolbetuximab plus first-line EOX (epirubicin, oxaliplatin, capecitabine; ZOL/EOX) significantly prolonged progression-free survival and overall survival in the FAST trial vs EOX alone. We report the patient-reported outcomes (PROs) of FAST in patients with advanced gastroesophageal adenocarcinoma.

**Methods:**

Patients were randomized to ZOL/EOX or EOX alone. Patients could receive ≤ 8 EOX cycles and remained on zolbetuximab until disease progression. PROs were collected using the EORTC QLQ-C30 and QLQ-STO22 before drug administration at day 1/cycle 1, day 1/cycle 5, end of EOX treatment, and q12w thereafter until disease progression. Time to deterioration (TTD), defined as the first meaningful worsening from baseline, in the individual QLQ-C30/QLQ-STO22 scores was analyzed. Longitudinal changes in scores from baseline were analyzed using a mixed-effects model for repeated measures (MMRM).

**Results:**

The per protocol population included 143 (ZOL/EOX: 69; EOX: 74) patients. Baseline QLQ-C30 and STO22 scores were comparable between arms and denoted intermediate-to-high quality of life (QoL), intermediate-to-low global health status (GHS) and low symptom burden. Descriptive analyses showed no differences between arms until end of EOX but maintenance therapy with zolbetuximab was associated with better QoL and less symptom burden thereafter. TTD for most scores favored ZOL/EOX over EOX and reached statistical significance for GHS (*p* = 0.008). MMRM results support TTD findings; no statistically significant differences were observed between arms in any score except for nausea and vomiting (*p* = 0.0181 favoring EOX).

**Conclusions:**

ZOL/EOX allowed patients to maintain good QoL and low symptom burden for longer than EOX alone.

**Supplementary Information:**

The online version contains supplementary material available at 10.1007/s10120-020-01153-6.

## Introduction

Gastric cancer is the fifth most commonly diagnosed cancer worldwide when excluding non-melanoma of skin, and together with liver cancer, the second most common cause of cancer-related deaths [1]. Gastroesophageal adenocarcinomas are more common in the elderly with a median age of 70 years at diagnosis worldwide [2]. Gastroesophageal adenocarcinomas are associated with poor survival, particularly at metastatic stages, with survival rates of < 5% at 5 years and a median overall survival shorter than 14 months [3]. Platinum-fluoropyrimidine-based chemotherapy is standard for first-line treatment for HER2-negative locally advanced unresectable and for metastatic stages [4–6].

Zolbetuximab is a first-in-class chimeric IgG1 monoclonal antibody that specifically binds to Claudin 18 splice variant 2 (CLDN18.2) on the cell surface and mediates cell death through antibody-dependent cellular cytotoxicity and complement-dependent cytotoxicity [7, 8]. CLDN18.2 is a highly selective gastric-lineage marker expressed in differentiated cells of the gastric mucosa, but not in any other normal human cell types [9, 10]. In normal gastric cells, CLDN18.2 exhibits a unique expression pattern that is restricted to the membrane, which includes a tight junctional region, of differentiated epithelial cells. Structure loss, which is one of the hallmarks of cancer, in the tumor gastric tissue may allow antibodies more accessible to CLDN18.2 [7, 11].

The efficacy and safety of zolbetuximab as add-on to first-line EOX (epirubicin, oxaliplatin, and capecitabine) has been assessed in the phase II FAST trial where zolbetuximab plus EOX showed a statistically significant benefit over EOX alone in the primary endpoint progression-free survival and key secondary endpoints overall survival and objective response rate [12]. As previously reported, FAST recruited patients with newly diagnosed advanced gastroesophageal adenocarcinomas whose tumors had ≥ 40% of tumor cells expressing CLDN18.2 with a moderate-to-strong (≥ 2 +) staining intensity using the CLAUDETECT™ 18.2 test and the 43-14A antibody clone [12]. In order to appropriately characterize the benefit-risk profile of first-line zolbetuximab, it is key to understand the patient’s perspective on the benefits of the treatment. Here we report the results of the patient-reported outcomes (PROs) collected in the FAST trial.

## Methods

### Study design and patients

Full details of the study design, patient eligibility criteria, and conduct of the study have been reported elsewhere [12]. Briefly, FAST (NCT01630083) is a phase II randomized study comparing the efficacy and safety of two different zolbetuximab dosing regimens as add-on to first-line EOX vs EOX alone in adults with histologically confirmed locally advanced inoperable, recurrent, or metastatic gastroesophageal adenocarcinoma positive for CLDN18.2 expression (defined as ≥ 40% of tumor cells with 2 + or 3 + staining intensity on CLAUDETECT™18.2 immunohistochemistry assay [9, 10]). Patients were recruited between July 2012 and June 2014.

Patients were randomly assigned 1:1 to receive zolbetuximab 800/600 mg/m^2^ plus EOX (ZOL/EOX) or EOX alone. The initial zolbetuximab dose was 800 mg/m^2^ as a 2-h IV infusion on day 1 of cycle 1, with 600 mg/m^2^ as a 2-h IV infusion administered on day 1 of every subsequent 21-day cycle. EOX consisted of epirubicin (50 mg/m^2^ as a 15-min IV infusion on day 1 of each cycle), oxaliplatin (130 mg/m^2^ as a 2-h IV infusion on day 1 of each cycle), and capecitabine (625 mg/m^2^ orally twice daily for 21 days in the morning and evening, starting with the evening of day 1 of each cycle). The standard EOX regimen was administered every 3 weeks for a maximum of 8 cycles, whereas zolbetuximab was continued until disease progression, withdrawal of consent, or unacceptable toxicity. At study conception, a third arm was planned to evaluate a potentiated zolbetuximab treatment regimen; however, a higher zolbetuximab dose was assessed in a third exploratory arm instead [12]. As reported elsewhere [12], the efficacy observed with the higher zolbetuximab dose was suboptimal compared with that of zolbetuximab 800/600 mg/m^2^ which is the dose currently assessed in the zolbetuximab phase III trials and the dose planned to be used in any further research. This publication focuses on the results of the ZOL/EOX and of the EOX alone arms. However, PRO data for the exploratory third arm (i.e., zolbetuximab 1000 mg/m^2^ plus EOX) are presented in the supplement for transparency (Table S6).

All procedures followed were in accordance with the ethical standards of the responsible committee on human experimentation (institutional and national) and with the Helsinki Declaration of 1964 and later versions. Informed consent to be included in the study was obtained from all patients.

### EORTC QLQ instruments and outcomes

In FAST, PROs were collected with the EORTC QLQ-C30 and its gastric cancer module (EORTC QLQ-ST22). The EORTC QLQ-C30 and STO22 responses were scored and analyzed in accordance with the scoring manual provided by the EORTC [13].

The EORTC QLQ-C30 is a cancer-specific 30-question instrument [14]. The questionnaire items are grouped into five functional scales (physical, role, cognitive, emotional, and social), three symptom scales (fatigue, pain, and nausea and vomiting), a global health status (GHS)/QoL scale, and single items (dyspnea, insomnia, appetite loss, constipation, diarrhea, and financial difficulties). Of the 30 items, 28 are scored on four-point Likert scales and the remaining two items (i.e., items 29 and 30 for GHS) are scored on modified seven-point linear analog scales. Higher scores on the functional and QoL scales translate to better health-related quality of life (HRQoL), whereas higher scores on the symptom scales translate to worse HRQoL.

The EORTC QLQ-STO22 takes into consideration 22 additional items related to gastric cancer [15]. This instrument includes five scales (dysphagia, chest and abdominal pain, reflux, eating restrictions, and anxiety) and four single items (dry mouth, body image, taste problems, and hair loss), reflecting disease symptoms, treatment side effects, and emotional issues, with higher scores indicating worse symptomatic problems. Higher scores on the symptoms denote worse HRQoL. The EORTC QLQ-STO22 was only administered in Russia, Ukraine, and Germany because no translations had been validated for Czech Republic, Bulgaria, and Latvia.

Both EORTC QLQ questionnaires were administered as paper versions and were completed before drug administration at day 1 of cycle 1, at day 1 cycle 5, at end of EOX treatment, and every 12 weeks thereafter until disease progression. Scores before drug administration at day 1 of cycle 1 are considered baseline scores.

### Statistical analyses

The PRO analysis was conducted on the full-analysis set (FAS) and the per-protocol analysis set (PPS). Results for both analysis sets were comparable. Results for the PPS are reported herein while results for the FAS are provided in the Supplement. The FAS included all patients randomized who received at least one dose of any study drug. The PPS comprised all patients without major protocol violations who received at least two complete cycles of therapy according to the protocol and had a second tumor evaluation after baseline. Completion rates at every assessment time point for both PRO instruments were defined as the number (percentage) of patients with evaluable forms completed among patients expected to have PRO assessments, e.g., who are alive and still on study.

The PRO analyses included descriptive statistics for observed domains and item scores and change from baseline per treatment arm and time point. No statistical analysis was conducted to assess differences in the means of descriptive statistics between treatment arms. For the median time to deterioration, 95% confidence intervals (CI) were calculated. Time to first deterioration was estimated using the Kaplan–Meier method and differences between arms were assessed using the log-rank test. Time to first deterioration was calculated as the time from the date of randomization to the date of the first clinically meaningful deterioration in the PRO score compared with the baseline score. The predetermined thresholds used to determine clinically meaningful deterioration are provided in Table S1 in the Supplement. Several groups have determined the meaningful within-group and within-patient important threshold for EORTC QLQ-C30 domains in different patient populations but none in patients with gastroesophageal cancer [16–18]. No thresholds have been reported for the EORTC STO22 instrument for this population either. Therefore, the meaningful within-patient important thresholds for these two instruments were determined based on the baseline distribution of the QLQ-C30 and QLQ-STO22 scores in FAST. A difference of at least one-half the baseline standard deviation was considered meaningful [19, 20]. Subjects who did not experience the first deterioration were censored at the date of the last instrument completion (i.e., date of the last non-missing value). Subjects with no baseline assessment were censored at the date of randomization.

To estimate longitudinal changes from baseline in PRO scores, a mixed model for repeated measures (MMRM) analysis was conducted. The model controlled for the treatment group, visit number, baseline score, and the interaction of these three covariates. The model included all data available and assumed that the missing observations were missing at random. For MMRM, cycles were converted into intervals to account for outlying visits and varying end of treatment measurement time points. Baseline corresponds to the PRO data collected prior to the first dose of therapy on day 1. The time intervals included in the MMRM were: interval 1, which spanned between day 1 and day 69 and encompassed the first four cycles of chemotherapy; interval 2 from day 70 to 110 (i.e., time of cycle 5 of chemotherapy with a window of 15 days); interval 3 from day 101 to 149; interval 4 from day 150 to 180 (i.e., planned time of the end of EOX with a window of 15 days); and interval 5, which spanned from day 181 until the last PRO collection (Figure S1 in Supplement). Data collected during the follow-up period, i.e., after the end of EOX treatment was analyzed using an ANCOVA (analysis of covariance) model fitted for LOCF (last observation carried forward) endpoint. The ANCOVA included treatment as the main effect. In both the MMRM and time to event analyses, an alpha of 0.05 was used as the cutoff for significance. Multiplicity adjustment was not applied.

A shift analysis was conducted to determine the percentage of patients who experienced a clinically important increase, no change, or a decrease in scores by the end of the EOX therapy. The shift analysis was conducted on the individual items of EORTC QLQ-C30 and STO22 for the PPS and also for the subgroup of the PPS with tumors expressing CLDN18.2 in ≥ 70% of tumor cells.

## Results

### Baseline characteristics and questionnaire compliance

We report the results for the ZOL/EOX (800/600) arm and the EOX alone arm in the PPS. The FAS population consisted of 161 patients (EOX: *n* = 84; ZOL/EOX: *n* = 77) and the PPS of 143 patients (EOX: *n* = 74; ZOL/EOX: *n* = 69). Demographics and baseline characteristics for patients with PRO data at baseline were well balanced between both treatment arms (Table 1).

**Table 1:** Demographics and baseline characteristics in FAST (PPS)

Characteristic	ZOL/EOX (*n* = 68)	EOX (*n* = 74)
Age, mean (SD)	57.0 (10.6)	55.5 (10.16)
Sex, *n* (%)		
Female	26 (38)	25 (34)
Male	42 (62)	49 (66)
ECOG, *n* (%)		
0	23 (34)	24 (32)
1	45 (66)	50 (68)
Location of primary tumor, *n* (%)		
Esophagus	2 (3)	3 (4)
GEJ	10 (15)	12 (16)
Stomach	56 (82)	59 (80)
Disease stage, *n* (%)		
Locally advanced	1 (1.5)	4 (5.4)
Metastatic	67 (98.5)	70 (94.6)
Metastatic sites, mean (SD)	3.12 (1.57)	3.14 (1.48)
Liver, *n* (%)	25 (36)	26 (35)
Lung, *n* (%)	13 (19)	13 (18)
Lymph node, *n* (%)	55 (81)	57 (77)
Histology at diagnosis, *n* (%)		
Diffuse	28 (41.2)	31 (41.9)
Intestinal	25 (36.8)	26 (35.1)
Mixed	9 (13.0)	9 (12.2)
Unknown	6 (8.8)	8 (10.8)
Peritoneal carcinomatosis, *n* (%)	17 (25)	21 (28)
CLDN18.2 expression ≥ 70%, n (%)	53 (78)	54 (73)
Previous gastrectomy, *n* (%)	21 (27.63)	23 (27.38)

*CLDN18.2* Claudin 18 splice variant 2, *ECOG* Eastern Cooperative Oncology Group, *EOX* epirubicin, oxaliplatin, and capecitabine, *GEJ* gastroesophageal junction, *HER2* human epidermal growth factor receptor 2, *PPS* per protocol set, *SD* standard deviation, *ZOL/EOX* zolbetuximab 800/600 mg/m^2^ plus EOX

A similar proportion of patients completed both instruments in the two arms up to cycle 8 of EOX. The proportion of patients who completed the EORTC QLQ-C30 at cycle 8 was 82% in the ZOL/EOX group and 77% in the EOX group. For the EORTC QLQ-STO22, this proportion was 66% and 60%, respectively. From the end of the EOX treatment onwards, the proportion of patients completing the questionnaire remained high in the ZOL/EOX arm but markedly decreased in the EOX arm (Table 2). This is consistent with longer progression-free survival with ZOL/EOX arm (7.5 months, 95% CI [5.6–11.3]) than with EOX alone (5.3 months, 95% CI [4.1–7.1]) and PROs being collected until disease progression only [12]. Exposure to the study drug was also longer in the ZOL/EOX arm where patients received a mean number of 10.8 cycles of zolbetuximab vs a mean of 5.5 EOX cycles in the EOX arm.

**Table 2:** EORTC QLQ-C30 and EORC QLQ-STO22 completion rates in FAST (PPS)

	EORTC QLQ-C30, % (*n*/*N*)		EORTC QLQ-STO22, % (*n*/*N*)	
	ZOL/EOX	EOX	ZOL/EOX	EOX
Cycle 1	98% (*n* = 67/68)	97% (*n* = 72/74)	78% (*n* = 53/68)	77% (*n* = 57/74)
Cycle 5	91% (*n* = 48/53)	86% (*n* = 49/57)	74% (*n* = 39/53)	72% (*n* = 41/57)
Cycle 8/EOT	82% (*n* = 55/67)	77% (*n* = 55/71)	66% (*n* = 44/67)	60% (*n* = 43/71)
Cycle 2 post EOX	82% (*n* = 23/28)	67% (*n* = 12/18)	64% (*n* = 18/28)	61% (*n* = 11/18)
Cycle 5 post EOX	84% (*n* = 16/19)	50% (*n* = 7/14)	63% (*n* = 12/19)	36% (*n* = 5/14)
Cycle 8 post EOX	93% (*n* = 13/14)	42% (*n* = 5/12)	57% (*n* = 8/14)	25% (*n* = 3/12)
Cycle 11 post EOX	92% (*n* = 12/13)	50% (*n* = 3/6)	61% (*n* = 8/13)	17% (*n* = 1/6)
Cycle 14 post EOX	83% (*n* = 10/12)	60% (*n* = 3/5)	58% (*n* = 7/12)	20% (*n* = 1/5)

*EORTC LQ-C30* European Organization for Research and Treatment of Cancer Quality of Life Questionnaire-Core 30, *EORTC QLQ-CTOO 22* European Organization for Research and Treatment of Cancer Quality of Life Questionnaire-gastric cancer module, *EOX* epirubicin, oxaliplatin, and capecitabine, *post EOX* assessment time after end of EOX treatment, *PPS* per protocol set, *ZOL/EOX* zolbetuximab 800/600 mg/m^2^ plus EOX

Baseline mean scores for EORC QLQ-C30 and STO22 were comparable between the two treatment arms (Table 3). In the ZOL/EOX arm, baseline scores suggest intermediate-to-high functioning, with scores ranging between 71.4 for role functioning and 91.3 for the cognitive functioning scale, and low symptom burden except for QLQ-C30 fatigue (38.4) and anxiety (60.7). In the EOX arm, baseline scores also suggest intermediate-to-high functioning, with scores ranging between 68.8 for role functioning and 86.0 for the cognitive functioning scale, and low symptom burden except for QLQ-C30 fatigue (43.3) and anxiety (63.2). In contrast, patients had low GHS/QoL with a mean score of 52.1 (range: 0–83.3) with ZOL/EOX and 49.9 (range: 0–91.7) with EOX.

**Table 3:** Baseline mean EORTC QLQ-C30 and EORTC QLQ-STO22 scores (PPS)

	Baseline score	
	ZOL/EOX	EOX
EORTC QLQ-C30		
Global health status	52.1 ± 17.74	49.9 ± 22.45
Physical functioning	76.7 ± 20.83	74.1 ± 22.45
Role functioning	71.4 ± 27.19	68.8 ± 28.65
Cognitive functioning	91.3 ± 14.92	86.0 ± 20.19
Emotional functioning	77.1 ± 19.29	71.0 ± 26.34
Social functioning	76.8 ± 22.81	72.1 ± 29.71
Appetite	28.9 ± 30.65	35.2 ± 32.31
Constipation	19.7 ± 28.03	21.9 ± 27.74
Diarrhea	6.2 ± 15.47	14.0 ± 25.82
Dyspnea	17.2 ± 24.28	14.1 ± 27.41
Fatigue	38.4 ± 20.76	43.3 ± 24.75
Financial difficulties	22.8 ± 27.32	28.5 ± 27.58
Insomnia	26.3 ± 28.35	29.6 ± 30.92
Nausea/vomiting	13.1 ± 18.61	15.0 ± 21.85
Pain	23.3 ± 24.44	26.4 ± 27.44
STO22		
STO22 total	59.0 ± 32.75	65.5 ± 32.39
Anxiety	60.7 ± 19.91	63.2 ± 25.12
Body image (symptom)	41.0 ± 32.75	34.5 ± 32.39
Body image (functioning)	59.0 ± 32.75	65.5 ± 32.39
Dry mouth	20.1 ± 28.75	19.9 ± 23.45
Dysphagia	12.8 ± 18.67	15.6 ± 19.34
Eating restrictions	22.5 ± 19.17	27.1 ± 22.47
Hair loss	35.4 ± 36.45	23.6 ± 33.68
Pain	22.1 ± 17.43	23.4 ± 17.54
Reflux	22.3 ± 18.05	18.7 ± 21.12
Taste	10.7 ± 21.46	12.9 ± 24.20

Data are given as mean ± standard deviation. *EORTC LQ-C30* European Organization for Research and Treatment of Cancer Quality of Life Questionnaire-Core 30, *EORTC QLQ-CTOO 22* European Organization for Research and Treatment of Cancer Quality of Life Questionnaire-gastric cancer module, *EOX* epirubicin, oxaliplatin, and capecitabine, *PPS* per protocol set, *ZOL/EOX* zolbetuximab 800/600 mg/m^2^ plus EOX

### Unadjusted change from baseline in HRQoL

**Fig. 1:**
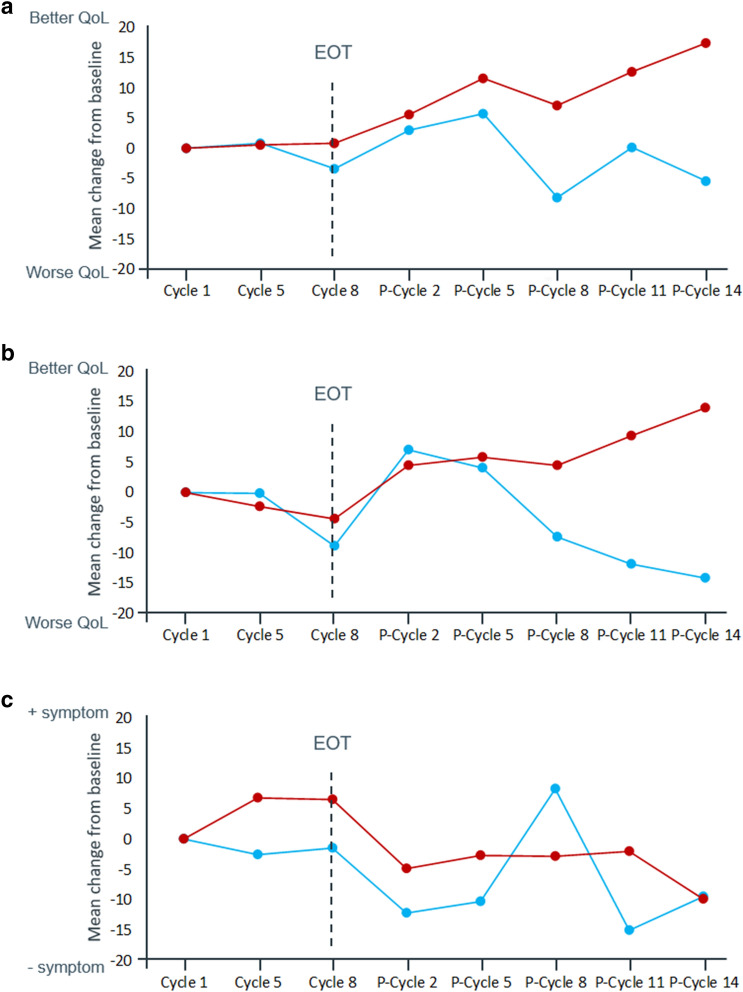
Descriptive analysis of mean change from baseline over time for **(a)** global health status/quality of life, **(b)** physical functioning, and **(c)** nausea/vomiting (PPS). Mean change from baseline throughout the study is provided for (a) global health status, (b) physical functioning, and (c) nausea/vomiting. Abbreviations: EOT: end of EOX treatment; EOX: epirubicin, oxaliplatin, and capecitabine; P-cycle: post end of EOX treatment cycle; PPS: per protocol set; ZOL/EOX: zolbetuximab 800/600 mg/m^2^ plus EOX

The unadjusted change from baseline results favored ZOL/EOX over EOX alone for GHS/QoL (Fig. 1a), and physical (Fig. 1b), role, and cognitive functioning. While no clinically meaningful changes from baseline were observed during EOX treatment in any arm, ZOL/EOX led to a marked progressive improvement after the end of EOX treatment vs an initial improvement followed by worsening with EOX. For emotional and social functioning, either no changes or a progressive improvement from baseline was observed for both arms but with improvement being greater with EOX alone. For symptoms, the general trend was either no change from baseline or improvement with ZOL/EOX vs worsening with EOX particularly after the end of EOX treatment. The only exception was nausea and vomiting. An increase in score from baseline was observed in the first eight cycles of EOX in the ZOL/EOX arm with no change from baseline thereafter. In contrast, no change from baseline was observed with EOX during the eight cycles of EOX with a decrease from baseline in most follow-up visits (Fig. 1c).

### Time to deterioration

**Fig. 2:**
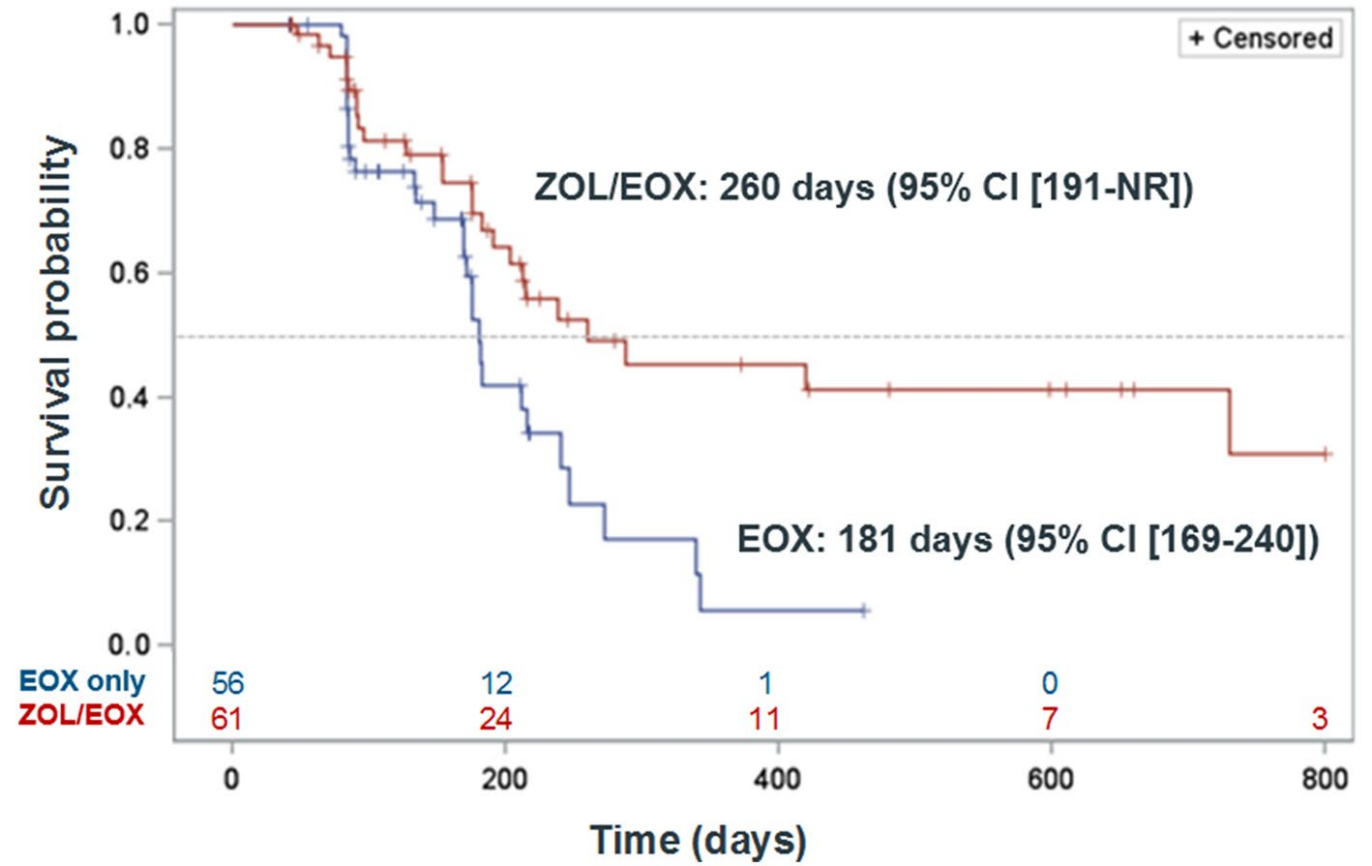
Kaplan–Meier curves of time to deterioration in HRQoL based on EORTC QLQ-C30 global health status/quality of life score (PPS). EORTC LQ-C30: European Organization for Research and Treatment of Cancer Quality of Life Questionnaire-Core 30; EOX: epirubicin, oxaliplatin, and capecitabine; HRQoL: health-related quality of life; PPS: per protocol set; ZOL/EOX: zolbetuximab 800/600 mg/m^2^ plus EOX

The ZOL/EOX combination significantly delayed deterioration in the QLQ-C30 GHS/QoL score by 2.6 months vs EOX alone (*p* = 0.008; Fig. 2). No statistically significant differences were observed for any other QLQ-C30 or QLQ-STO22 domains or items including nausea and vomiting (*p* = 0.4802). However, the median time to deterioration consistently favored ZOL/EOX (i.e., longer median time) for all items except for body image, which was numerically longer for EOX (188 vs 171 days). No numerical difference was observed for pain (QLQ-C30; 288 days for both arms) and reflux (185 vs 184; Table S1 in Supplement).

### Adjusted change from baseline in HRQoL

Several clinically meaningful changes from baseline were observed in the ZOL/EOX arm at different time points. The addition of zolbetuximab led to a clinically meaningful worsening from baseline of cognitive functioning at interval 4, of nausea and vomiting at intervals 2 and 4, and of taste troubles at interval 4 and overall. In contrast, ZOL/EOX improved anxiety from baseline at interval 2 (Table S2 in Supplement).

Several clinically meaningful changes from baseline were also observed in the EOX arm at several time points. EOX alone was associated with a clinically meaningful worsening from baseline of financial troubles at interval 5, of dry mouth at intervals 2 and 5 and overall, and of taste troubles at intervals 4 and 5 and overall. In contrast, EOX improved anxiety from baseline at interval 2 (Table S2 in Supplement).

**Fig. 3:**
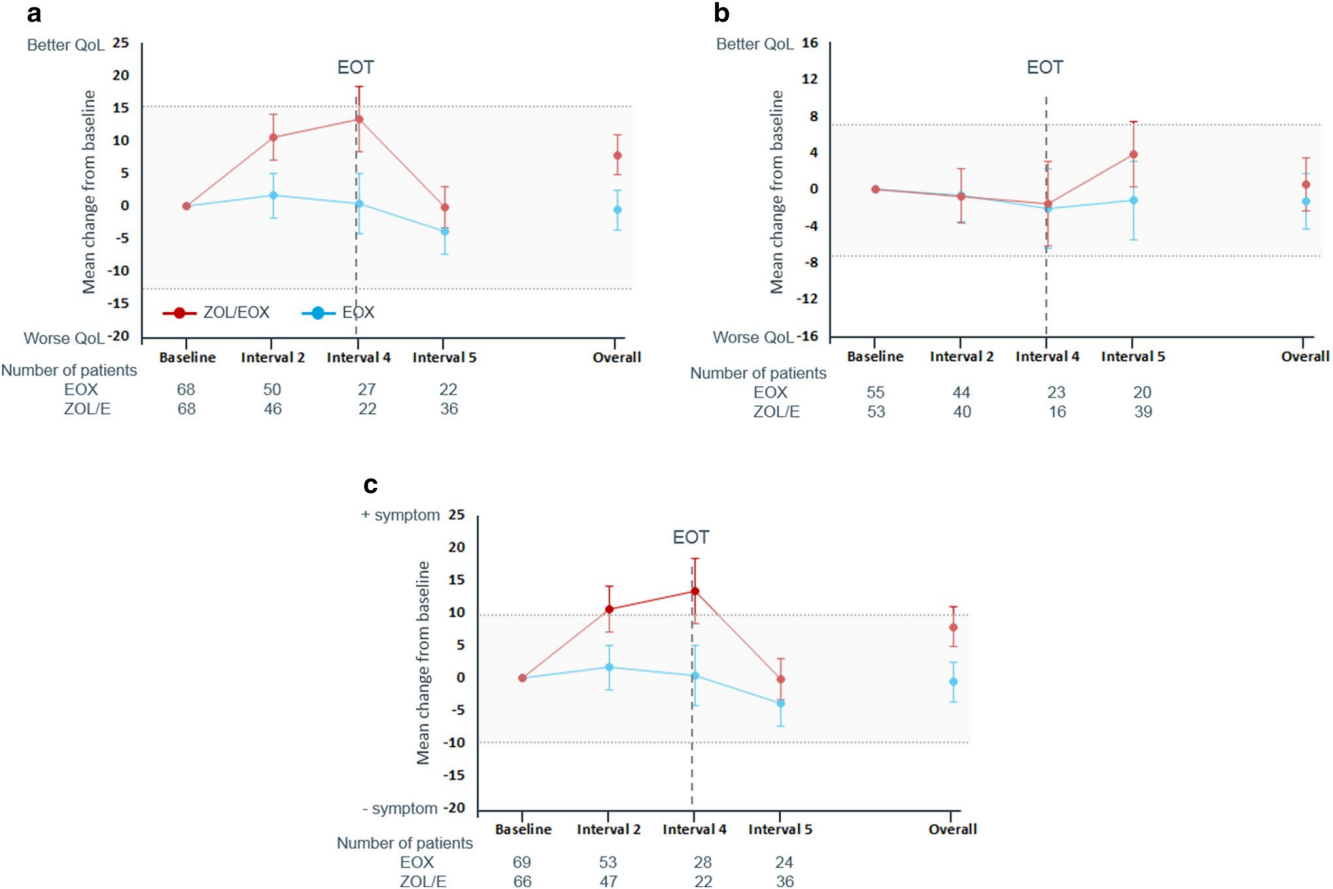
Change from baseline in **(a)** global health status/quality of life, **(b)** EORTC QLQ-STO22 total score, and **(c)** nausea/vomiting (MMRM analysis; PPS). Data are provided as LS mean and standard error change from baseline for (a) the EORTC QLQ-C30 global health status, (b) the EORTC QLQ-STO22 total score, and (c) nausea/vomiting. Abbreviations: EORTC LQ-C30: European Organization for Research and Treatment of Cancer Quality of Life Questionnaire-Core 30; EORTC QLQ-STO22: European Organization for Research and Treatment of Cancer Quality of Life Questionnaire-gastric cancer module; EOT: end of EOX treatment; EOX: epirubicin, oxaliplatin, and capecitabine; Interval 2: time of cycle 5 of EOX with a window of 15 days; Interval 4: planned time of the end of EOX treatment with a window of 15 days); interval 5: from the end of EOX treatment until the last PRO collection; MMRM: mixed model for repeated measures; PPS: per protocol set; ZOL/EOX: zolbetuximab 800/600 mg/m^2^ plus EOX

When comparing both treatment arms, the results of the MMRM analyses confirm the unadjusted change from baseline and time to deterioration findings with a trend towards better HRQoL and less symptoms in the ZOL/EOX arm vs EOX alone. No statistically significant differences were observed between the ZOL/EOX and EOX arms throughout the overall study period for GHS/QoL (*p* = 0.5837; Fig. 3a), total STO22 score (*p* = 0.7170; Fig. 3b), or any functioning scales or symptoms except for nausea/vomiting (Table S2 in Supplement). Change from baseline in the EORTC QLQ-C30 nausea/vomiting score (Fig. 3c) at interval 2 (difference: − 8.866, 95% CI [− 16.739, − 0.992]; *p* = 0.0277), interval 4 (difference: − 12.934, 95% CI [− 25.671, − 0.197]; *p* = 0.0467) and overall (difference: − 8.480, 95% CI [− 15.470, − 1.490]; *p* = 0.0181) favored EOX vs ZOL/EOX. However, only differences at interval 4 were clinically meaningful. No clinically or statistically significant differences were observed between treatment arms for pain in the stomach area (EORTC QLQ-STO22; Table S2 in Supplement) or anywhere (EORTC QLQ-C30; Table S2 in Supplement) but the change from baseline numerically favored ZOL/EOX vs EOX after EOX completion (i.e., at interval 5).

### Response shift

**Fig. 4:**
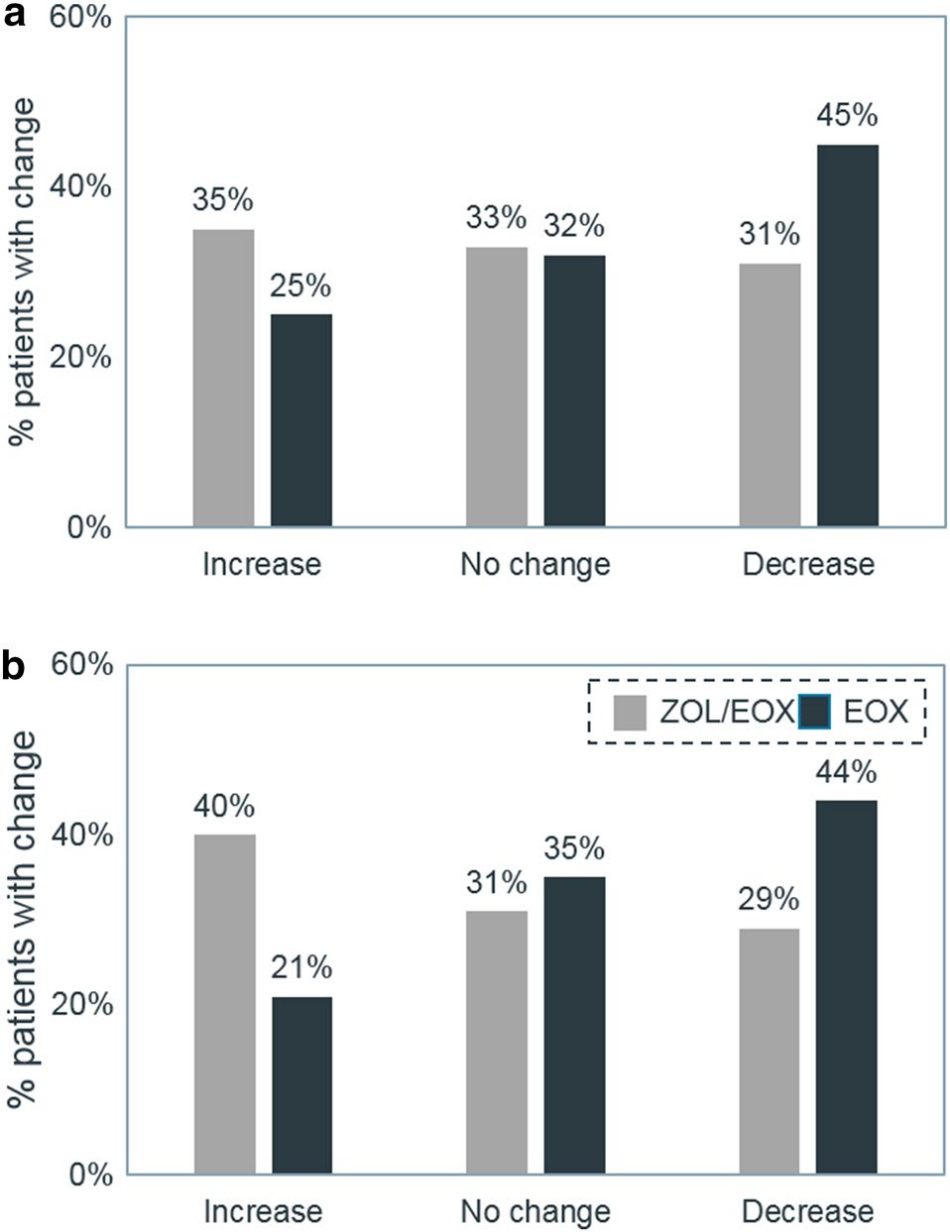
Change from baseline at cycle 8 in the overall HRQoL in the **(a)** overall FAST population (PPS) and **(b)** the ≥ 70% CLDN18.2 expressing patients. CLDN18.2: Claudin 18 splice variant 2; EOX: epirubicin, oxaliplatin, and capecitabine; HRQoL: health-related quality of life; PPS: per protocol set; ZOL/EOX: zolbetuximab 800/600 mg/m^2^ plus EOX

Response shift was assessed at the single item level. Statistically significant differences between arms were observed for several items by cycle 8. In all cases, the results favored ZOL/EOX, with a higher proportion of patients experiencing a decrease in the given symptom, or an increase in overall HRQoL. The items for which statistical significance was reached were overall QoL in the past week (Fig. 4a), trouble belching, feeling tense, pain interference with activities, acid indigestion and burning, and shortness of breath. The benefit observed for ZOL/EOX over EOX was greater for the patient subgroup with high CLDN18.2 expression, i.e., in patients with tumors with 70% or more gastric cancer cells expressing CLDN18.2, particularly for overall QoL in the past week (Fig. 4b).

## Discussion

In the FAST study, the addition of zolbetuximab to EOX significantly prolonged median progression-free survival and median overall survival in the overall patient population and the subgroup of patients with tumors expressing CLDN18.2 in ≥ 70% of tumor cells [12]. No significant safety concerns were identified with the addition of zolbetuximab to first-line EOX [12]. The manageable safety profile of zolbetuximab is further supported by the PRO results which overall, favored ZOL/EOX over EOX alone particularly after end of EOX treatment when a trend towards improving HRQoL and reducing the symptom burden from baseline was observed in the ZOL/EOX arm with maintenance therapy with zolbetuximab.

The only domain for which the results tended to favor EOX over ZOL/EOX was the score for nausea and vomiting. While no significant differences were observed between arms for time to clinically meaningful deterioration, worsening in nausea and vomiting score from baseline was statistically greater with ZOL/EOX than with EOX alone at interval 2 (*p* = 0.0277), interval 4 (*p* = 0.0467) and overall (*p* = 0.0181) and reached clinical meaningfulness at interval 4. Nausea and vomiting have been reported as adverse events associated with single-agent zolbetuximab [10]. These symptoms are likely on-target effects due to zolbetuximab’s pharmacodynamics, namely CLDN18.2 expression in normal cells being restricted to the stomach mucosa [10]. Nausea and vomiting are also frequently observed with EOX [21]. The impact of nausea and vomiting on QoL was not analyzed in FAST. However, several studies have shown a negative impact of nausea and vomiting in the context of chemotherapy even for patients receiving moderately emetic chemotherapy [22]. Greater burden related to nausea and vomiting observed in the ZOL/EOX arm during EOX treatment (interval 2) and at EOX treatment completion (interval 4) vs the EOX arm may have adversely impacted QoL in the ZOL/EOX arm. However, the significant delay in time to deterioration of GHS/QoL score with ZOL/EOX compared to EOX alone suggests otherwise,

It cannot be ruled out that the toxicity of the chemotherapy backbone (i.e., EOX) may have offset clinically meaningful or statistically significant improvement of HRQoL with zolbetuximab. This is supported by the descriptive analyses (unadjusted change from baseline) which consistently favored ZOL/EOX vs EOX alone for the post EOX end of treatment assessments. This is also further supported by MMRM analysis for interval 5, which corresponds to the post-EOX study period. Although no statistically significant differences between treatment arms were observed for any PRO score at interval 5, the majority of LS means favored ZOL/EOX. EOX is a triplet with high efficacy [23], but the use of triplet over doublet regimens has been questioned because triplets, while having similar efficacy, have higher toxicity than doublet regimens [24, 25]. The toxicity of EOX may also account for the different HRQoL-related findings between the FAST and ToGA trials. Unlike trastuzumab, which significantly delayed time to deterioration of all EORTC QLQ-C30 and STO22 scales vs chemotherapy in the ToGA trial [26], zolbetuximab only significantly delayed the time to deterioration of GHS/QoL compared with EOX alone. These differences may be due to the comparator being a doublet (cisplatin plus either capecitabine or fluorouracil) in ToGA vs a triplet in FAST, differences in the time to deterioration definition (first unconfirmed event in FAST vs definitive deterioration in ToGA), and more stringent threshold in FAST than in ToGA where a change in 10 units was applied as the threshold to all scales with a change in five units in the sensitivity analysis. In addition, the percentage of patients with a diffuse histology type was markedly higher in the FAST trial (41 vs 9% in ToGA). Diffuse histology type is associated with the worst prognosis among all histology types of gastric cancer with rapid progression and short survival [27].

Zolbetuximab is currently being assessed in two phase III trials with two different first-line comparators, both doublets. NCT03504397 will compare the efficacy of zolbetuximab plus mFOLFOX6 (oxaliplatin, leucovorin, and fluorouracil) vs placebo plus mFOLFOX6 and NCT03653507 will compare zolbetuximab plus CAPOX (oxaliplatin plus capecitabine) vs placebo plus CAPOX. Both studies are recruiting subjects with CLDN18.2-positive, HER2-negative, locally advanced unresectable or metastatic gastroesophageal adenocarcinoma. Patient-reported outcomes will be collected in both studies using the EORTC QLQ C30, EORTC QLQ-Gastric Module 25 (QLQ-OG25), the EORTC-QLQ-STO22 Belching subscale, Global Pain, and the EQ-5D. These two studies will provide additional information on the impact of zolbetuximab on patients’ HRQoL.

This study has several limitations. The add-on setting and the expected different duration of the EOX and zolbetuximab therapies rendered a blinded study design difficult. While the limitation of the open-label nature of PROs collection in FAST cannot be discarded, this limitation is mitigated by the fact that the control arm (EOX) was an active therapy and considered part of the standard of care. In addition, the study included several objective endpoints (e.g., progression-free survival and overall survival), which are less prone to bias in open-label studies and which showed clear superiority for the experimental intervention. In this context, despite the caveats of the open-label nature, it seems appropriate to conclude that ZOL/EOX did not adversely influence HRQoL.

Another limitation is the marked decrease in the number of patients completing the PRO instruments after the end of EOX treatment in both arms. While the number of patients completing the questionnaires remained high during EOX treatment, this number dropped rapidly in both arms thereafter (ZOL/EOX: *n* = 7–23; EOX: *n* = 1–12).

## Conclusions

The results from FAST suggest that the addition of zolbetuximab to EOX does not adversely impact HRQoL compared with EOX alone; this reflects the tolerability of zolbetuximab. In addition, ZOL/EOX led to greater HRQoL and lower symptom burden than EOX alone after the end of the EOX treatment. Patients who have not yet received chemotherapy have relatively good functioning and HRQoL with low symptom burden. Maintaining good HRQoL and low symptom burden is important in oncology in general and becomes essential in cancers like gastroesophageal where survival is very short.

## Supplementary Information

Below is the link to the electronic supplementary material.

Supplementary file1 (PDF 368 KB)
