# Supplementary material for: Patient-reported outcomes from the phase II FAST trial of zolbetuximab plus EOX compared to EOX alone as first-line treatment of patients with metastatic CLDN18.2+ gastroesophageal adenocarcinoma
Source: Gastric Cancer. 2021 Mar 23;24(3):721–30. doi: 10.1007/s10120-020-01153-6 (PMC8064997; doi:10.1007/s10120-020-01153-6)
Supplement: Supplementary file 1 — Supplementary file1 (PDF 368 KB) [file 10120_2020_1153_MOESM1_ESM.pdf]

Supplement

Table S1 Median time to deterioration for EORTC QLQ-C30 and EORTC QLQ-STO22 (PPS)

		Median time to deterioration (PPS)		
**Domain**	**MID**	**ZOL/EOX**	**EOX**	**P-value**
		**Median [95% CI], days**	**Median [95% CI], days**	
**EORTC QLQ-C30**				
Global Score	10.1	260 [191-NR]	181 [169-240]	**0.0084**
Physical functioning	10.8	212 [154-NR]	170 [133-181]	0.0833
Role functioning	13.9	175 [100-636]	169 [125-187]	0.7108
Social functioning	13.2	231 [96-636]	182 [169-246]	0.5484
Emotional functioning	11.7	351 [177-NR]	246 [168-339]	0.2037
Cognitive functioning	9.0	199 [112-NR]	171 [133-336]	0.6361
Appetite loss	15.8	344 [218-NR]	279 [168-NR]	0.4614
Constipation	13.9	NR [191-NR]	NR	0.6390
Diarrhea	10.9	NR [589-NR]	NR	0.1066
Dyspnea	12.9	NR [466-NR]	NR	0.4016
Fatigue	11.5	610 [288-NR]	NR [176-NR]	0.4141
Financial difficulties	13.8	NR [291-NR]	NR	0.5179
Insomnia	14.8	596 [274-NR]	NR [188-NR]	0.8537
Nausea and vomiting	10.2	NR [416-NR]	NR [429-NR]	0.4802
Pain (EORTC-QLQ C30)	13.0	288 [153-NR]	288 [91-NR]	0.7602
**EORTC QLQ-STO22**				

		Median time to deterioration (PPS)		
**Domain**	**MID**	**ZOL/EOX Median [95% CI], days**	**EOX Median [95% CI], days**	**P-value**
STO22 total	7.6	NR [222-NR]	NR [208-NR]	0.8382
Anxiety	11.3	344 [183-733]	210 [93-NR]	0.1899
Body image (symptom)	16.3	291 [222-446]	NR [169-NR]	0.9798
Body image (function)	16.3	171 [96-NR]	188 [133-NR]	0.9354
Dry mouth	13.0	NR [282-NR]	NR	0.3606
Dysphagia	9.5	NR [176-NR]	429 [168-NR]	0.9720
Eating restrictions	10.5	NR [218-NR]	188 [106-429]	0.0521
Hair loss	17.6	NR	NR [84-NR]	0.3980
Pain (STO22)	8.7	NR [217-NR]	342 [176-NR]	0.3805
Reflux	9.8	174 [88-191]	175 [166-NR]	0.2153
Trouble taste	11.4	NR	NR	0.1873

Abbreviations: MID: minimal clinically important difference; NR: not yet reached; PPS: per protocol set; ZOL/EOX: zolbetuximab 800/600 mg/m^2 plus EOX.

Table S2 Change from baseline at intervals 2, 4, and 5 (MMRM; PPS)

		Interval 2 (time of cycle 5 of EOX ±15 days)		Interval 4 (planned time of EOT ±15 days)		Interval 5 (from EOT until the last PRO collection)		Overall	
		LS mean (SE)/[95% CI]	p-value	LS mean (SE)/[95% CI]	p-value	LS mean (SE)/[95% CI]	p-value	LS mean (SE)/[95% CI]	p-value
**EORTC QLQ-C30**									
Physical functioning	ZOL/E	-2.301 (3.140)	0.4653	-6.080 (4.054)	0.1387	0.773 (3.503)	0.8259	-2.536 (3.000)	0.4008
	EOX	-1.074 (3.136)	0.7327	-6.827 (4.075)	0.0983	-6.830 (4.075)	0.0978	-4.910 (3.246)	0.1344
	Difference	1.227 [-5.496, 7.950]	0.7181	-0.747 [-10.822, 9.327]	0.8823	-7.603 [-16.811, 1.605]	0.1039	-2.374 [-9.251, 4.502]	0.4933
Role functioning	ZOL/E	-0.269 (4.370)	0.9511	-10.284 (5.947)	0.0882	-0.657 (4.755)	0.8904	-3.737 (4.216)	0.3779
	EOX	-1.345 (4.389)	0.7598	**-12.036 (5.962)**	**0.0472**	**-11.589 (5.643)**	**0.0436**	-8.323 (4.522)	0.0690
	Difference	-1.077 [-10.641, 8.488]	0.8237	-1.752 [-16.883, 13.379]	0.8174	-10.932 [-23.783, 1.920]	0.0941	-4.587 [-14.421, 5.247]	0.3561
Cognitive functioning	ZOL/E	-2.405 (3.269)	0.4633	**-13.778 (4.132)**	**0.0014**	-3.290 (3.261)	0.3167	**-6.491 (3.036)**	**0.0360**
	EOX	-4.325 (3.138)	0.1709	-7.303 (3.916)	0.0669	-5.337 (3.651)	0.1485	-5.655 (3.075)	0.0702
	Difference	-1.919 [-8.752, 4.913]	0.5783	6.476 [-3.542, 16.493]	0.1997	-2.047 [-10.295, 6.201]	0.6214	0.836 [-5.774, 7.446]	0.8010
Emotional functioning	ZOL/E	1.254 (3.358)	0.7094	-2.889 (4.736)	0.5444	-1.546 (3.989)	0.6994	-1.060 (3.096)	0.7331
	EOX	0.434 (3.242)	0.8938	-2.706 (4.548)	0.5541	-5.979 (4.830)	0.2200	-2.750 (3.293)	0.4064
	Difference	-0.821 [-7.637, 5.996]	0.8115	0.183 [-11.882, 12.247]	0.9758	-4.433 [-15.632, 6.766]	0.4313	-1.690 [-8.651, 5.270]	0.6293
Social functioning	ZOL/E	-3.446 (4.238)	0.4177	-3.028 (5.651)	0.5941	0.124 (4.777)	0.9793	-2.117 (3.976)	0.5961
	EOX	3.048 (4.141)	0.4633	-2.015 (5.501)	0.7153	-4.334 (5.569)	0.4388	-1.101 (4.211)	0.7946
	Difference	6.494 [-2.148, 15.136]	0.1391	1.013 [-13.127, 15.154]	0.8860	-4.458 [-17.296, 8.379]	0.4902	1.016 [-7.898, 9.930]	0.8207
Fatigue	ZOL/E	2.438 (3.613)	0.5011	**11.478 (5.322)**	**0.0357**	-0.415 (4.447)	0.9258	4.500 (3.533)	0.2072
	EOX	-0.230 (3.675)	0.9501	4.704 (5.248)	0.3737	5.817 (5.412)	0.2858	3.430 (3.875)	0.3789
	Difference	-2.669 [-10.337, 5.000]	0.4915	-6.774 [-20.460, 6.912]	0.3244	6.233 [-6.149, 18.615]	0.3182	-1.070 [-9.375, 7.235]	0.7977
Pain	ZOL/E	-2.336 (3.996)	0.5599	9.820 (5.757)	0.0933	-2.753 (4.798)	0.5676	1.577 (4.066)	0.6992
	EOX	-2.428 (3.931)	0.5381	6.552 (5.668)	0.2518	6.044 (5.480)	0.2734	3.389 (4.306)	0.4333

		Interval 2 (time of cycle 5 of EOX ±15 days)		Interval 4 (planned time of EOT ±15 days)		Interval 5 (from EOT until the last PRO collection)		Overall	
		**LS mean (SE)/[95% CI]**	**p-value**	**LS mean (SE)/[95% CI]**	**p-value**	**LS mean (SE)/[95% CI]**	**p-value**	**LS mean (SE)/[95% CI]**	**p-value**
	Difference	-0.092 [-7.969, 7.786]	0.9816	-3.268 [-17.933, 11.396]	0.6563	8.797 [-3.739, 21.333]	0.1657	1.812 [-7.358, 10.983]	0.6948
Nausea/Vomiting	ZOL/E	**10.419 (3.527)**	**0.0037**	**13.234 (5.022)**	**0.0105**	-0.269 (3.107)	0.9312	**7.795 (3.105)**	**0.0137**
	EOX	1.554 (3.367)	0.6452	0.300 (4.656)	0.9488	-3.910 (3.555)	0.2754	-0.686 (3.116)	0.8263
	Difference	**-8.866 [-16.739, -0.992]**	**0.0277**	**-12.934 [-25.671, -0.197]**	**0.0467**	-3.641 [-11.532, 4.249]	0.3592	**-8.480 [-15.470, -1.490]**	**0.0181**
Appetite	ZOL/E	-4.332 (4.659)	0.3544	0.255 (6.447)	0.9686	-9.003 (5.507)	0.1058	-4.360 (4.535)	0.3392
	EOX	-0.901 (4.665)	0.8472	2.810 (6.354)	0.6596	-3.929 (6.383)	0.5399	-0.673 (4.841)	0.8897
	Difference	3.431 [-6.468, 13.330]	0.4931	2.555 [-13.700, 18.811]	0.7538	5.074 [-9.561, 19.708]	0.4910	3.687 [-6.615, 13.988]	0.4782
Constipation	ZOL/E	**-9.888 (4.172)**	**0.0193**	1.317 (5.586)	0.8144	-7.617 (4.920)	0.1257	-5.396 (3.885)	0.1688
	EOX	**-11.062 (4.007)**	**0.0067**	-6.738 (5.395)	0.2162	-2.475 (5.808)	0.6712	-6.758 (4.126)	0.1056
	Difference	-1.174 [-9.562, 7.214]	0.7817	-8.055 [-22.110, 5.999]	0.2550	5.141 [-8.357, 18.639]	0.4489	-1.363 [-10.064, 7.339]	0.7556
Diarrhea	ZOL/E	3.167 (4.080)	0.4390	3.914 (4.353)	0.3724	3.079 (4.515)	0.4973	3.387 (3.312)	0.3096
	EOX	**8.569 (3.961)**	**0.0325**	6.020 (4.230)	0.1595	7.502 (5.315)	0.1629	**7.364 (3.509)**	**0.0393**
	Difference	5.402 [-3.597, 14.401]	0.2362	2.106 [-8.452, 12.664]	0.6902	4.423 [-8.207, 17.053]	0.4857	3.977 [-3.279, 11.233]	0.2784
Insomnia	ZOL/E	1.726 (3.951)	0.6630	**11.704 (5.491)**	**0.0364**	5.088 (4.797)	0.2923	6.173 (3.883)	0.1152
	EOX	-2.010 (3.883)	0.6058	4.274 (5.163)	0.4100	5.667 (5.632)	0.3178	2.644 (4.002)	0.5104
	Difference	-3.736 [-12.211, 4.739]	0.3836	-7.429 [-20.841, 5.982]	0.2723	0.579 [-12.719, 13.877]	0.9309	-3.529 [-12.313, 5.255]	0.4267
Dyspnea	ZOL/E	2.816 (4.013)	0.4841	7.051 (5.394)	0.1973	-0.571 (3.881)	0.8836	3.099 (3.584)	0.3907
	EOX	5.938 (3.956)	0.1362	5.513 (5.152)	0.2889	3.509 (4.513)	0.4399	4.987 (3.773)	0.1911
	Difference	3.121 [-5.783, 12.026]	0.4883	-1.538 [-15.157, 12.080]	0.8211	4.080 [-5.986, 14.146]	0.4198	1.888 [-6.282, 10.058]	0.6456
Financial	ZOL/E	6.901 (5.237)	0.1900	8.254 (5.848)	0.1628	8.383 (5.398)	0.1250	7.846 (4.681)	0.0980
	EOX	2.584 (5.173)	0.6184	7.517 (5.615)	0.1853	**17.224 (6.117)**	**0.0064**	9.108 (4.858)	0.0651
	Difference	-4.317 [-15.159, 6.524]	0.4310	-0.736 [-14.243, 12.770]	0.9132	8.841 [-4.916, 22.597]	0.2032	1.262 [-8.582, 11.107]	0.7991
GHS/QoL	ZOL/E	-0.778 (2.959)	0.7931	-1.652 (4.598)	0.7206	3.746 (3.527)	0.2917	0.439 (2.909)	0.8805

		Interval 2 (time of cycle 5 of EOX ±15 days)		Interval 4 (planned time of EOT ±15 days)		Interval 5 (from EOT until the last PRO collection)		Overall	
		**LS mean (SE)/[95% CI]**	**p-value**	**LS mean (SE)/[95% CI]**	**p-value**	**LS mean (SE)/[95% CI]**	**p-value**	**LS mean (SE)/[95% CI]**	**p-value**
	EOX	-0.755 (2.898)	0.7949	-2.152 (4.339)	0.6214	-1.254 (4.264)	0.7697	-1.387 (3.021)	0.6472
	Difference	0.023 [-6.056, 6.101]	0.9941	-0.501 [-12.057, 11.055]	0.9311	-5.000 [-15.017, 5.017]	0.3216	-1.826 [-8.434, 4.782]	0.5837
**EORTC QLQ-STO22**									
Body image	ZOL/E	-5.200 (6.742)	0.4425	-14.059 (8.367)	0.0985	-1.866 (7.728)	0.8101	-7.042 (6.276)	0.2660
	EOX	-1.041 (6.547)	0.8740	-5.239 (7.764)	0.5023	-6.709 (9.120)	0.4651	-4.330 (6.481)	0.5066
	Difference	4.159 [-9.797, 18.114]	0.5546	8.821 [-10.526, 28.167]	0.3628	-4.843 [-26.069, 16.383]	0.6479	2.712 [-10.769, 16.193]	0.6889
Dysphagia	ZOL/E	-4.737 (3.459)	0.1742	0.710 (5.559)	0.8990	0.967 (3.984)	0.8094	-1.020 (3.688)	0.7831
	EOX	-1.182 (3.502)	0.7365	5.896 (4.901)	0.2356	0.777 (4.667)	0.8685	1.830 (3.745)	0.6270
	Difference	3.554 [-3.418, 10.527]	0.3135	5.186 [-8.115, 18.488]	0.4313	-0.190 [-10.739, 10.359]	0.9712	2.850 [-5.251, 10.952]	0.4806
Pain	ZOL/E	0.533 (3.295)	0.8719	4.471 (4.900)	0.3651	0.552 (4.124)	0.8941	1.852 (3.325)	0.5792
	EOX	-1.076 (3.281)	0.7437	3.338 (4.323)	0.4425	3.193 (4.913)	0.5184	1.818 (3.371)	0.5914
	Difference	-1.609 [-8.413, 5.194]	0.6390	-1.133 [-12.549, 10.284]	0.8427	2.641 [-8.796, 14.078]	0.6444	-0.034 [-7.184, 7.117]	0.9925
Reflux	ZOL/E	-3.741 (3.001)	0.2155	-2.199 (3.759)	0.5610	-4.222 (2.926)	0.1545	-3.387 (2.649)	0.2071
	EOX	-0.556 (2.952)	0.8510	-4.109 (3.560)	0.2539	-4.301 (3.371)	0.2067	-2.989 (2.796)	0.2899
	Difference	3.184 [-3.359, 9.728]	0.3356	-1.910 [-11.273, 7.453]	0.6828	-0.079 [-7.697, 7.539]	0.9835	0.399 [-5.601, 6.398]	0.8944
Eating restrictions	ZOL/E	2.950 (3.822)	0.4421	7.013 (4.473)	0.1235	0.600 (4.403)	0.8920	3.521 (3.505)	0.3195
	EOX	-2.618 (3.816)	0.4945	-3.195 (4.198)	0.4504	-1.521 (5.151)	0.7688	-2.445 (3.741)	0.5161
	Difference	-5.568 [-13.721, 2.584]	0.1778	-10.208 [-20.560, 0.144]	0.0531	-2.121 [-14.184, 9.941]	0.7252	-5.966 [-13.706, 1.775]	0.1283
Anxiety	ZOL/E	**-11.782 (4.319)**	**0.0076**	-7.912 (6.363)	0.2197	-6.429 (4.487)	0.1574	**-8.707 (4.070)**	**0.0358**
	EOX	**-12.152 (4.319)**	**0.0061**	-9.512 (5.681)	0.0993	-9.514 (5.291)	0.0774	**-10.393 (4.230)**	**0.0166**
	Difference	-0.371 [-9.751, 9.010]	0.9375	-1.601 [-16.880, 13.678]	0.8334	-3.086 [-15.056, 8.884]	0.6066	-1.686 [-10.701, 7.330]	0.7103
Dry mouth	ZOL/E	5.813 (5.434)	0.2874	12.203 (7.227)	0.0975	7.428 (6.408)	0.2512	8.481 (5.259)	0.1119
	EOX	**15.313 (5.474)**	**0.0063**	12.315 (6.651)	0.0694	**18.616 (7.545)**	**0.0165**	**15.415 (5.553)**	**0.0073**

		Interval 2 (time of cycle 5 of EOX ±15 days)		Interval 4 (planned time of EOT ±15 days)		Interval 5 (from EOT until the last PRO collection)		Overall	
		**LS mean (SE)/[95% CI]**	**p-value**	**LS mean (SE)/[95% CI]**	**p-value**	**LS mean (SE)/[95% CI]**	**p-value**	**LS mean (SE)/[95% CI]**	**p-value**
	Difference	9.500 [-1.924, 20.924]	0.1019	0.112 [-17.019, 17.244]	0.9895	11.188 [-5.854, 28.231]	0.1930	6.934 [-4.608, 18.475]	0.2340
Taste	ZOL/E	9.201 (5.543)	0.1002	**21.808 (7.891)**	**0.0081**	7.509 (5.899)	0.2083	**12.839 (5.440)**	**0.0219**
	EOX	7.758 (5.637)	0.1723	**15.328 (7.230)**	**0.0394**	**13.457 (6.678)**	**0.0485**	**12.181 (5.674)**	**0.0363**
	Difference	-1.443 [-13.206, 10.321]	0.8079	-6.480 [-25.558, 12.598]	0.4955	5.948 [-8.873, 20.769]	0.4239	-0.658 [-12.664, 11.347]	0.9127
Hair loss	ZOL/E	2.950 (3.822)	0.4421	7.013 (4.473)	0.1235	0.600 (4.403)	0.8920	3.521 (3.505)	0.3195
	EOX	-2.618 (3.816)	0.4945	-3.195 (4.198)	0.4504	-1.521 (5.151)	0.7688	-2.445 (3.741)	0.5161
	Difference	-5.568 [-13.721, 2.584]	0.1778	-10.208 [-20.560, 0.144]	0.0531	-2.121 [-14.184, 9.941]	0.7252	-5.966 [-13.706, 1.775]	0.1283
Total STO	ZOL/E	0.881 (2.727)	0.7474	6.067 (3.627)	0.0998	0.596 (3.252)	0.8553	2.514 (2.684)	0.3522
	EOX	0.151 (2.712)	0.9557	2.788 (3.335)	0.4064	1.368 (3.853)	0.7239	1.436 (2.818)	0.6121
	Difference	-0.730 [-6.343, 4.884]	0.7965	-3.278 [-11.669, 5.113]	0.4346	0.772 [-8.145, 9.688]	0.8626	-1.079 [-7.001, 4.844]	0.7170

Abbreviations: EOT: end of EOX therapy; GHS: global health sate; MMRM: mixed model for repeated measures; ZOL/EOX: zolbetuximab 800/600 mg/m^2 plus EOX.

Table S3 Baseline mean EORTC QLQ-C30 and EORTC QLQ-STO22 scores (FAS)

	Baseline score	
	ZOL/EOX	EOX
**EORTC QLQ-C30**		
Global health status	51.9 ± 17.2	49.8 ± 21.6
Physical wellbeing	76.5 ± 20.7	74.7 ± 22.0
Role wellbeing	71.3 ± 27.6	70.0 ± 28.2
Cognitive wellbeing	91.5 ± 14.6	86.5 ± 20.3
Emotional wellbeing	77.3 ± 18.9	70.5 ± 26.2
Social wellbeing	76.8 ± 23.4	73.6 ± 28.9
Appetite	28.9 ± 29.7	34.6 ± 31.8
Constipation	18.9 ± 27.1	24.2 ± 29.5
Diarrhea	5.9 ± 15.0	13.1 ± 24.7
Dyspnea	17.6 ± 25.4	14.4 ± 26.3
Fatigue	38.0 ± 21.6	42.9 ± 24.7
Financial difficulties	23.0 ± 26.2	29.5 ± 28.7
Insomnia	26.6 ± 28.7	30.9 ± 30.9
Nausea/Vomiting	12.8 ± 18.2	15.0 ± 20.7
Pain	24.2 ± 24.5	26.8 ± 26.5
**STO22**		
STO22 total	59.5 ± 19.6	62.6 ± 24.5
Anxiety	37.8 ± 32.4	32.8 ± 32.0
Body image (symptom)	18.9 ± 27.7	20.5 ± 23.3
Body image (functioning)	62.1 ± 32.4	67.2 ± 32.0
Dry mouth	11.8 ± 17.8	15.2 ± 19.2
Dysphagia	21.3 ± 18.7	26.4 ± 23.8
Eating restrictions	59.0 ± 35.4	69.0 ± 23.1

	Baseline score	
	ZOL/EOX	EOX
Hair loss	22.2 ± 17.5	23.8 ± 17.2
Pain	21.2 ± 17.5	19.1 ± 21.8
Reflux	10.0 ±20.6	12.5 ± 23.4
Taste	62.1 ± 32.4	67.2 ± 32.0

Data are given as mean ± standard deviation. Abbreviations: CWB: cognitive well-being; EORTC LQ-C30: European Organization for Research and Treatment of Cancer Quality of Life Questionnaire-Core 30; EORTC QLQ-CTOO 22: European Organization for Research and Treatment of Cancer Quality of Life Questionnaire-gastric cancer module; EOX: epirubicin, oxaliplatin, and capecitabine; FAS: full analysis set; ZOL/EOX: zolbetuximab 800/600 mg/m^2 plus EOX.

Table S4 Median time to deterioration for EORTC QLQ-C30 and EORTC QLQ-STO22 (FAS)

		Median time to deterioration (FAS)		
**Domain**	**MID**	**ZOL/EOX Median [95% CI], days**	**EOX Median [95% CI], days**	**P-value**
**EORTC QLQ-C30**				
Global Score	10.1	260 [191-NR]	181 [169-216]	0.0100
Physical functioning	10.8	212 [154-NR]	170 [133-181]	0.0867
Role functioning	13.9	175 [100-636]	168 [125-187]	0.2460
Social functioning	13.2	231 [96-636]	182 [169-339]	0.5637
Emotional functioning	11.7	351 [177-NR]	246 [168-339]	0.2108
Cognitive functioning	9.0	199 [112-NR]	181 [133-336]	0.6548
Appetite loss	15.8	344 [218-NR]	279 [168-NR]	0.4710
Constipation	13.9	NR [191-NR]	NR [NR-NR]	0.6264
Diarrhea	10.9	NR [589-NR]	NR [NR-NR]	0.0708
Dyspnea	12.9	NR [466-NR]	NR [NR-NR]	0.5432
Fatigue	11.5	610 [288-NR]	NR [176-NR]	0.4228
Financial difficulties	13.8	NR [291-NR]	NR [NR-NR]	0.6712
Insomnia	14.8	596 [274-NR]	NR [188-NR]	0.8658
Nausea and vomiting	10.2	NR [416-NR]	NR [429-NR]	0.4857
Pain (EORTC-QLQ C30)	13.0	288 [153-NR]	288 [91-NR]	0.7826
**EORTC QLQ-STO22**				
STO22 total	7.6	NR [222-NR]	NR [208-NR]	0.8579

		Median time to deterioration (FAS)		
Domain	MID	ZOL/EOX<br>Median [95% CI], days	EOX<br>Median [95% CI], days	P-value
Anxiety	11.3	344 [183-733]	210 [93-NR]	0.1970
Body image (symptom)	16.3	291 [222-466]	NR [169-NR]	0.9581
Body image (functioning)	16.3	171 [96-NR]	188 [133-NR]	0.9560
Dry mouth	13.0	NR [282-NR]	NR [NR-NR]	0.3534
Dysphagia	9.5	NR [176-NR]	429 [168-NR]	0.9969
Eating restrictions	10.5	NR [218-NR]	188 [106-429]	0.0553
Hair loss	17.6	NR [NR-NR]	NR [84-NR]	0.3980
Pain (STO22)	8.7	NR [217-NR]	342 [176-NR]	0.3897
Reflux	9.8	174 [88-191]	175 [166-NR]	0.2063
Trouble taste	11.4	NR [NR-NR]	NR [NR-NR]	0.1917

Abbreviations: FAS: full analysis set; MID: minimal clinically important difference; NR: not yet reached; ZOL/EOX: zolbetuximab 800/600 mg/m^2 plus EOX.

Table S5 Change from baseline at intervals 2, 4, and 5 (MMRM; FAS)

		Interval 2 (time of cycle 5 of EOX ±15 days)		Interval 4 (planned time of EOT ±15 days)		Interval 5 (from EOT until the last PRO collection)		Overall	
		**LS mean (SE)/[95% CI]**	**p-value**	**LS mean (SE)/[95% CI]**	**p-value**	**LS mean (SE)/[95% CI]**	**p-value**	**LS mean (SE)/[95% CI]**	**p-value**
**EORTC QLQ-C30**									
Physical functioning	ZOL/E	-2.326 (3.126)	0.4583	-6.096 (4.047)	0.1371	0.743 (3.495)	0.8323	-2.560 (2.990)	0.3948
	EOX	-1.082 (3.105)	0.7282	-6.842 (4.065)	0.0967	-6.857 (4.063)	0.0955	-4.927 (3.227)	0.1309
	Difference	1.244 [-5.418, 7.905]	0.7118	-0.746 [-10.810, 9.317]	0.8823	-7.600 [-16.791, 1.592]	0.1034	-2.367 [-9.213, 4.478]	0.4927
Role functioning	ZOL/E	-0.323 (4.350)	0.9410	-10.353 (5.936)	0.0856	-0.714 (4.747)	0.8808	-3.797 (4.203)	0.3689
	EOX	-1.414 (4.339)	0.7451	**-12.127 (5.936)**	**0.0446**	**-11.659 (5.629)**	**0.0419**	-8.400 (4.492)	0.0647
	Difference	-1.091 [-10.560, 8.377]	0.8195	-1.774 [-16.870, 13.321]	0.8147	-10.944 [-23.782, 1.893]	0.0933	-4.603 [-14.387, 5.181]	0.3520
Cognitive functioning	ZOL/E	-2.442 (3.258)	0.4549	**-13.792 (4.126)**	**0.0014**	-3.315 (3.258)	0.3126	**-6.516 (3.030)**	**0.0349**
	EOX	-4.163 (3.110)	0.1834	-7.173 (3.904)	0.0709	-5.292 (3.646)	0.1513	-5.543 (3.060)	0.0744
	Difference	-1.721 [-8.491, 5.049]	0.6150	6.619 [-3.378, 16.615]	0.1892	-1.977 [-10.220, 6.266]	0.6332	0.974 [-5.608, 7.555]	0.7683
Emotional functioning	ZOL/E	1.305 (3.373)	0.6995	-2.878 (4.738)	0.5461	-1.501 (4.000)	0.7086	-1.025 (3.107)	0.7425
	EOX	0.865 (3.243)	0.7902	-2.568 (4.556)	0.5751	-5.741 (4.842)	0.2400	-2.481 (3.302)	0.4549
	Difference	-0.440 [-7.266, 6.387]	0.8985	0.310 [-11.754, 12.374]	0.9590	-4.239 [-15.455, 6.976]	0.4522	-1.456 [-8.430, 5.517]	0.6780
Social functioning	ZOL/E	-3.389 (4.251)	0.4268	-3.021 (5.654)	0.5952	0.153 (4.789)	0.9746	-2.086 (3.987)	0.6024
	EOX	3.574 (4.137)	0.3896	-1.797 (5.508)	0.7453	-4.004 (5.577)	0.4751	-0.742 (4.217)	0.8608
	Difference	6.963 [-1.678, 15.604]	0.1130	1.225 [-12.912, 15.362]	0.8624	-4.156 [-17.011, 8.699]	0.5205	1.344 [-7.578, 10.266]	0.7646
Fatigue	ZOL/E	2.414 (3.608)	0.5049	**11.469 (5.315)**	**0.0356**	-0.440 (4.444)	0.9213	4.481 (3.528)	0.2085
	EOX	-0.553 (3.651)	0.8800	4.550 (5.239)	0.3887	5.591 (5.403)	0.3040	3.196 (3.861)	0.4104
	Difference	-2.966 [-10.597, 4.665]	0.4424	-6.919 [-20.584, 6.746]	0.3136	6.031 [-6.339, 18.400]	0.3336	-1.285 [-9.564, 6.995]	0.7576
Pain	ZOL/E	-2.274 (4.012)	0.5719	9.851 (5.760)	0.0924	-2.700 (4.800)	0.5752	1.626 (4.073)	0.6908
	EOX	-1.874 (3.931)	0.6344	6.997 (5.663)	0.2208	6.467 (5.472)	0.2408	3.863 (4.302)	0.3715

		Interval 2 (time of cycle 5 of EOX ±15 days)		Interval 4 (planned time of EOT ±15 days)		Interval 5 (from EOT until the last PRO collection)		Overall	
		**LS mean (SE)/[95% CI]**	**p-value**	**LS mean (SE)/[95% CI]**	**p-value**	**LS mean (SE)/[95% CI]**	**p-value**	**LS mean (SE)/[95% CI]**	**p-value**
	Difference	0.399 [-7.487, 8.285]	0.9202	-2.853 [-17.507, 11.800]	0.6973	9.167 [-3.350, 21.684]	0.1483	2.238 [-6.920, 11.396]	0.6278
Nausea/Vomiting	ZOL/E	**10.394 (3.513)**	**0.0036**	**13.208 (5.015)**	**0.0105**	-0.289 (3.105)	0.9261	**7.771 (3.097)**	**0.0138**
	EOX	1.492 (3.333)	0.6551	0.247 (4.641)	0.9577	-3.939 (3.553)	0.2717	-0.733 (3.098)	0.8135
	Difference	-8.902 [-16.702, -1.102]	0.0257	**-12.961 [-25.669, -0.253]**	**0.0458**	-3.650 [-11.539, 4.239]	0.3580	**-8.504 [-15.459, -1.550]**	**0.0172**
Appetite	ZOL/E	-4.299 (4.657)	0.3578	0.329 (6.444)	0.9594	-8.954 (5.502)	0.1073	-4.308 (4.531)	0.3445
	EOX	-1.283 (4.641)	0.7827	2.632 (6.341)	0.6793	-4.169 (6.372)	0.5147	-0.940 (4.823)	0.8459
	Difference	3.016 [-6.846, 12.878]	0.5452	2.303 [-13.938, 18.544]	0.7773	4.785 [-9.836, 19.405]	0.5155	3.368 [-6.905, 13.641]	0.5158
Constipation	ZOL/E	**-9.828 (4.153)**	**0.0194**	1.375 (5.579)	0.8061	-7.550 (4.913)	0.1285	-5.334 (3.875)	0.1725
	EOX	**-11.011 (3.971)**	**0.0065**	-6.683 (5.387)	0.2192	-2.411 (5.799)	0.6789	-6.702 (4.110)	0.1070
	Difference	-1.184 [-9.491, 7.124]	0.7779	-8.058 [-22.100, 5.984]	0.2544	5.139 [-8.353, 18.631]	0.4489	-1.368 [-10.041, 7.306]	0.7540
Diarrhea	ZOL/E	3.074 (4.103)	0.4552	3.918 (4.357)	0.3723	2.959 (4.525)	0.5151	3.317 (3.326)	0.3216
	EOX	**7.954 (3.967)**	**0.0472**	5.815 (4.239)	0.1748	7.214 (5.324)	0.1801	6.995 (3.519)	0.0506
	Difference	4.881 [-4.155, 13.916]	0.2861	1.897 [-8.668, 12.463]	0.7196	4.255 [-8.387, 16.897]	0.5028	3.678 [-3.599, 10.954]	0.4552
Insomnia	ZOL/E	1.767 (3.933)	0.6540	**11.741 (5.478)**	**0.0354**	5.151 (4.790)	0.2858	6.220 (3.870)	0.1113
	EOX	-2.043 (3.844)	0.5962	4.255 (5.137)	0.4097	5.711 (5.625)	0.3135	2.641 (3.977)	0.5083
	Difference	-3.810 [-12.210, 4.590]	0.3702	-7.486 [-20.858, 5.887]	0.2673	0.560 [-12.733, 13.852]	0.9331	-3.579 [-12.323, 5.166]	0.4182
Dyspnea	ZOL/E	2.689 (4.026)	0.5054	6.900 (5.390)	0.2067	-0.670 (3.899)	0.8642	2.973 (3.592)	0.4111
	EOX	5.324 (3.948)	0.1803	5.042 (5.147)	0.3313	3.167 (4.530)	0.4873	4.511 (3.773)	0.2364
	Difference	2.635 [-6.264, 11.534]	0.5582	-1.858 [-15.448, 11.732]	0.7843	3.837 [-6.268, 13.941]	0.4496	1.538 [-6.626, 9.702]	0.7076
Financial	ZOL/E	6.839 (5.223)	0.1928	8.173 (5.847)	0.1668	8.308 (5.388)	0.1277	7.774 (4.671)	0.1003
	EOX	2.732 (5.138)	0.5959	7.562 (5.601)	0.1815	**17.258 (6.104)**	**0.0062**	9.184 (4.835)	0.0616
	Difference	-4.107 [-14.891, 6.676]	0.4514	-0.611 [-14.101, 12.879]	0.9279	8.950 [-4.775, 22.675]	0.1968	1.411 [-8.397, 11.219]	0.7752
GHS/QoL	ZOL/E	-0.789 (2.957)	0.7900	-1.655 (4.596)	0.7199	3.748 (3.529)	0.2917	0.435 (2.908)	0.8815

		Interval 2 (time of cycle 5 of EOX ±15 days)		Interval 4 (planned time of EOT ±15 days)		Interval 5 (from EOT until the last PRO collection)		Overall	
		LS mean (SE)/[95% CI]	p-value	LS mean (SE)/[95% CI]	p-value	LS mean (SE)/[95% CI]	p-value	LS mean (SE)/[95% CI]	p-value
	EOX	-1.034 (2.882)	0.7205	-2.393 (4.329)	0.5821	-1.335 (4.268)	0.7555	-1.587 (3.013)	0.5996
	Difference	-0.244 [-6.291, 5.802]	0.9362	-0.738 [-12.277, 10.800]	0.8984	-5.083 [-15.108, 4.942]	0.3141	-2.022 [-8.615, 4.571]	0.5432
**EORTC QLQ-STO22**									
Body image	ZOL/E	-5.424 (6.777)	0.4255	-14.275 (8.392)	0.0945	-1.978 (7.704)	0.7983	-7.226 (6.288)	0.2547
	EOX	-2.124 (6.535)	0.7459	-6.005 (7.770)	0.4425	-7.246 (9.075)	0.4281	-5.125 (6.465)	0.4309
	Difference	3.300 [-10.691, 17.291]	0.6398	8.270 [-11.136, 27.677]	0.3947	-5.268 [-26.371, 15.835]	0.6174	2.101 [-11.358, 15.560]	0.7560
Dysphagia	ZOL/E	-4.649 (3.442)	0.1801	0.777 (5.536)	0.8890	1.015 (3.971)	0.7994	-0.952 (3.668)	0.7961
	EOX	-0.982 (3.457)	0.7770	6.073 (4.857)	0.2179	0.908 (4.646)	0.8458	2.000 (3.704)	0.5913
	Difference	3.667 [-3.233, 10.566]	0.2936	5.296 [-7.929, 18.521]	0.4192	-0.107 [-10.622, 10.408]	0.9837	2.952 [-5.077, 10.981]	0.4613
Pain	ZOL/E	0.556 (3.277)	0.8656	4.498 (4.889)	0.3611	0.568 (4.118)	0.8908	1.874 (3.312)	0.5731
	EOX	-0.970 (3.236)	0.7651	3.454 (4.291)	0.4234	3.266 (4.902)	0.5080	1.916 (3.339)	0.5678
	Difference	-1.526 [-8.251, 5.199]	0.6526	-1.044 [-12.416, 10.329]	0.8544	2.698 [-8.731, 14.126]	0.6371	0.043 [-7.059, 7.144]	0.9905
Reflux	ZOL/E	-3.657 (2.985)	0.2234	-2.124 (3.756)	0.5742	-4.155 (2.922)	0.1605	-3.312 (2.642)	0.2159
	EOX	-0.436 (2.911)	0.8813	-4.025 (3.552)	0.2625	-4.216 (3.361)	0.2142	-2.892 (2.777)	0.3023
	Difference	3.221 [-3.255, 9.697]	0.3251	-1.901 [-11.258, 7.457]	0.6842	-0.062 [-7.670, 7.546]	0.9871	0.420 [-5.556, 6.395]	0.8884
Eating restrictions	ZOL/E	2.995 (3.801)	0.4326	7.051 (4.461)	0.1205	0.662 (4.392)	0.8806	3.570 (3.490)	0.3108
	EOX	-2.586 (3.762)	0.4936	-3.163 (4.177)	0.4526	-1.460 (5.139)	0.7773	-2.403 (3.711)	0.5198
	Difference	-5.582 [-13.638, 2.475]	0.1718	-10.214 [-20.543, 0.114]	0.0525	-2.122 [-14.170, 9.925]	0.7248	-5.973 [-13.668, 1.722]	0.1257
Anxiety	ZOL/E	**-11.671 (4.314)**	**0.0081**	-7.765 (6.347)	0.2271	-6.361 (4.480)	0.1611	**-8.599 (4.064)**	**0.0378**
	EOX	**-11.619 (4.273)**	**0.0079**	-9.160 (5.658)	0.1107	-9.269 (5.278)	0.0844	**-10.016 (4.204)**	**0.0200**
	Difference	0.052 [-9.258, 9.363]	0.9911	-1.394 [-16.637, 13.848]	0.8543	-2.907 [-14.851, 9.036]	0.6267	-1.416 [-10.391, 7.558]	0.7538
Dry mouth	ZOL/E	5.888 (5.405)	0.2787	12.253 (7.210)	0.0954	7.484 (6.392)	0.2465	8.542 (5.237)	0.1079
	EOX	**15.532 (5.403)**	**0.0051**	12.455 (6.620)	0.0651	**18.779 (7.517)**	**0.0153**	**15.589 (5.507)**	**0.0063**

		Interval 2 (time of cycle 5 of EOX ±15 days)		Interval 4 (planned time of EOT ±15 days)		Interval 5 (from EOT until the last PRO collection)		Overall	
		**LS mean (SE)/[95% CI]**	**p-value**	**LS mean (SE)/[95% CI]**	**p-value**	**LS mean (SE)/[95% CI]**	**p-value**	**LS mean (SE)/[95% CI]**	**p-value**
	Difference	9.644 [-1.658, 20.946]	0.0934	0.202 [-16.892, 17.296]	0.9811	11.295 [-5.718, 28.307]	0.1881	7.047 [-4.427, 18.521]	0.2239
Taste	ZOL/E	9.164 (5.521)	0.1002	**21.795 (7.875)**	**0.0080**	7.500 (5.878)	0.2073	12.819 (5.422)	0.0216
	EOX	7.376 (5.571)	0.1890	**15.114 (7.203)**	**0.0413**	13.209 (6.640)	0.0513	**11.900 (5.628)**	**0.0390**
	Difference	-1.788 [-13.449, 9.873]	0.7611	-6.680 [-25.723, 12.362]	0.4816	5.710 [-9.049, 20.469]	0.4407	-0.919 [-12.856, 11.017]	0.8777
Hair loss	ZOL/E	2.995 (3.801)	0.4326	7.051 (4.461)	0.1205	0.662 (4.392)	0.8806	3.570 (3.490)	0.3108
	EOX	-2.586 (3.762)	0.4936	-3.163 (4.177)	0.4526	-1.460 (5.139)	0.7773	-2.403 (3.711)	0.5198
	Difference	-5.582 [-13.638, 2.475]	0.1718	-10.214 [-20.543, 0.114]	0.0525	-2.122 [-14.170, 9.925]	0.7248	-5.973 [-13.668, 1.722]	0.1257
Total STO	ZOL/E	0.975 (2.723)	0.8631	6.147 (3.623)	0.0952	0.672 (3.246)	0.8368	2.598 (2.681)	0.3359
	EOX	0.465 (2.686)	0.7211	3.050 (3.319)	0.3618	1.567 (3.845)	0.6851	1.694 (2.801)	0.5474
	Difference	-0.510 [-6.090, 5.069]	0.8560	-3.097 [-11.479, 5.284]	0.4597	0.895 [-8.010, 9.801]	0.8407	-0.904 [-6.807, 4.999]	0.7605

Abbreviations: EOT: end of EOX therapy; FAS: full analysis set; GHS: global health sate; MMRM: mixed model for repeated measures; ZOL/EOX: zolbetuximab 800/600 mg/m^2 plus EOX.

Table S6 Change from baseline over time in the EORTC QLQ-C30 and EORC QLQ-STO22 scores in the exploratory third arm

	Change from baseline at:	MID	FAS			PPS		
			ZOL+EOX (1000)	ZOL+EOX (800/600)	EOX alone	ZOL+EOX (1000)	ZOL+EOX (800/600)	EOX alone
**EORTC QLQ-C30**								
Appetite loss	Cycle 5	15.8	0	-1.8	-2.6	-0.1	-1.8	-3.2
	EOT		-2.6	2.6	-0.7	-3.1	3.2	-0.7
	LOCF		-2.7	2.9	1.9	-3.2	3.4	1.8
Cognitive functioning	Cycle 5	9.0	-1.6	-4.2	-0.3	-2.4	-4	0.3
	EOT		-6.2	-6.7	-2.9	-6.2	-6.8	-2.6
	LOCF		-6.4	-8.7	-5	-6.6	-8.8	-4.7
Constipation	Cycle 5	13.9	-7.6	-5.1	-12.1	-7	-5.9	-9.8
	EOT		-2	-1	-7.5	-2.4	-1.5	-4.9
	LOCF		-4.1	-1.7	-3.6	-4.4	-2.2	-0.9
Diarrhea	Cycle 5	10.9	0.1	6.4	4.6	0.3	6.2	3.7
	EOT		1.7	6.6	0.6	-0.2	6.6	-0.1
	LOCF		1.2	5.6	0.7	-0.4	5.5	0
Dyspnea	Cycle 5	12.9	-0.3	-3.1	1.2	-0.1	-2.7	1.5
	EOT		2.5	0.8	8.8	2.6	1.6	9.5
	LOCF		1.4	1.7	7.5	1.5	2.4	8.1
Emotional functioning	Cycle 5	11.7	2.1	1.3	6	2.4	1.5	5.6
	EOT		1.1	-0.7	2.1	1.9	-0.9	1.5
	LOCF		2.5	-4.7	-2.7	3.3	-5	-3.3
Fatigue	Cycle 5	11.5	-0.5	1.3	-2.9	0.5	0.9	-3.3
	EOT		9.8	7.8	3.5	9.9	7	3.6
	LOCF		8.2	5.2	6.4	8.2	4.5	6.4

	Change from baseline at:	MID	FAS			PPS		
			ZOL+EOX (1000)	ZOL+EOX (800/600)	EOX alone	ZOL+EOX (1000)	ZOL+EOX (800/600)	EOX alone
Financial difficulties	Cycle 5	13.8	1.9	3.8	-4	1.3	4.1	-3
	EOT		-1.5	4.4	2	-2.1	5.2	2.4
	LOCF		-2.2	3	4.9	-2.8	-2.5	5.4
Nausea & vomiting	Cycle 5	10.2	4.6	7.1	-2.5	4.8	6.8	-2.5
	EOT		1.9	8.3	-0.4	2.2	6.6	-0.5
	LOCF		-1.7	5.9	2.7	-1.4	4.4	2.7
Pain	Cycle 5	13.0	-7.9	-6.8	-7.5	-8.5	-5.9	-7.1
	EOT		0.3	0.9	5	-1.8	0.5	5.3
	LOCF		-0.6	1.3	4.3	-2.6	1	4.7
Physical functioning	Cycle 5	10.8	-2.3	-2.2	-0.7	-2.8	-2.4	-0.1
	EOT		-8.2	-3.8	-9.1	-8.8	-4.4	-8.9
	LOCF		-8	-4.1	-11.9	-8.6	-4.7	-11.7
Global score	Cycle 5	10.1	2.8	0.6	0.9	3.2	0.4	0.8
	EOT		-3.9	0.8	-3.3	-3.4	1	-3.1
	LOCF		-4.3	-1	-6.6	-3.9	-0.9	-6.6
Role functioning	Cycle 5	13.9	1.6	2.1	-1.6	2	2	-0.4
	EOT		-4.1	-4.9	-11.1	-3.6	-5.7	-10.3
	LOCF		-5.4	-3.9	-14	-5	-4.5	-13.2
Social functioning	Cycle 5	13.2	4.5	-2.9	4.1	4.2	-2.9	5.6
	EOT		-6.7	-2.1	-0.7	-6.6	-2.6	0.3
	LOCF		-5.2	-3.4	-6.9	-5.1	-3.8	-6
Insomnia	Cycle 5	14.8	-8.4	-1.6	-11.2	-8.5	-1.3	-9.9
	EOT		-1.1	0.1	3	-3	0.9	4.9
	LOCF		-2.1	0.4	2.9	-3.8	1.1	4.8

	Change from baseline at:	MID	FAS			PPS		
			ZOL+EOX (1000)	ZOL+EOX (800/600)	EOX alone	ZOL+EOX (1000)	ZOL+EOX (800/600)	EOX alone
EORTC QLQ-ST022								
Body image (functioning)	Cycle 5	16.3	4.6	-3.1	-0.5	6.3	0	1.2
	EOT		3.3	-2.8	-11.1	5	-0.7	-8.9
	LOCF		4.5	-3	-8.7	6.2	-0.7	-6.5
Body image (symptom)	Cycle 5	16.3	-4.6	3.2	0.5	-6.3	0	-1.2
	EOT		-3.3	2.9	11.1	-5	0.7	8.9
	LOCF		-4.5	3.1	8.7	-6.3	0.7	6.5
Anxiety	Cycle 5	11.3	-2.1	-9.4	-10.2	-2.5	-10.6	-10.8
	EOT		-1.7	-3.1	-4.5	-2.6	-4	-5.1
	LOCF		-2.2	0.2	-3.5	-3	-0.7	-4.1
Dry mouth	Cycle 5	13.0	1.7	3.9	12.8	2.8	2.7	13.4
	EOT		7.2	4.8	9.7	7.3	4.1	10.3
	LOCF		3.2	5.6	16	3.3	4.9	16.6
Dysphagia	Cycle 5	9.5	-1.1	-3	-3.3	-2.1	-4	-3.7
	EOT		-0.8	-1.4	1.7	-1.5	-2.2	1.4
	LOCF		-1.8	1	6.2	-2.6	0.2	6
Eating restrictions	Cycle 5	10.5	1	0.3	-9.1	0.9	-0.9	-9.8
	EOT		3	2	-6.6	3.3	1.2	-7
	LOCF		0.9	3.7	-4.8	1.1	2.8	-5.3
Hair loss	Cycle 5	17.6	24.5	20	23.4	23.3	20	22.9
	EOT		12.3	12.1	17.6	11	12.1	16.8
	LOCF		11.6	12.3	14.3	10.3	12.3	13.4
Pain	Cycle 5	8.7	-5	-1.3	-7.5	-5.4	-1.2	-7.1
	EOT		-0.8	0.9	-0.7	-2.4	0.6	-0.3

	Change from baseline at:	MID	FAS			PPS		
			ZOL+EOX (1000)	ZOL+EOX (800/600)	EOX alone	ZOL+EOX (1000)	ZOL+EOX (800/600)	EOX alone
	LOCF		-1.7	0.9	0.2	-3.1	-36.2	0.6
Reflux	Cycle 5	9.8	1.8	-9.2	-6.4	1.5	-10.3	-6
	EOT		1.1	-7.6	-6.5	-0.3	-8.9	-6
	LOCF		0	-6.8	-1.3	-1.3	-8.1	-0.8
Trouble taste	Cycle 5	11.4	14.4	11.4	11.1	14.7	10.7	10.7
	EOT		13.4	12.7	10	13.4	12.6	10.1
	LOCF		9.3	12.4	12.7	9.2	12.2	12.7

Abbreviations: EOT: end of EOX therapy; FAS: full analysis set; LOCF: last observation carried forward; MID: minimal clinically important difference; PPS: per protocol set; ZOL+EOX (800/600): zolbetuximab 800/600 mg/m^2 plus EOX; ZOL+EOX (1000): zolbetuximab 1000 mg/m^2 plus EOX.

Figure S1 Schedule of treatment and MMRM analysis in FAST

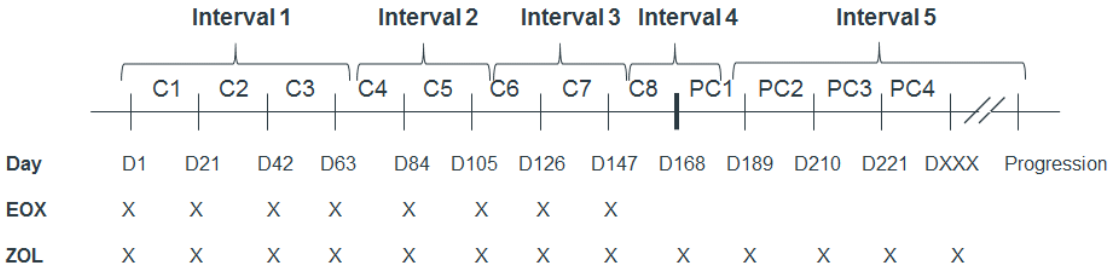

Abbreviations: DX: day number X; CX: EOX cycle number X; PCX: zolbetuximab cycles after end of EOX therapy; ZOL: zolbetuximab. End of EOX therapy is illustrated with a thick vertical line on day 168.

Figure S2 Descriptive analysis of mean change from baseline over time for (a) Global Health Status/Quality of life, (b) physical functioning, and (c) nausea/vomiting (FAS)

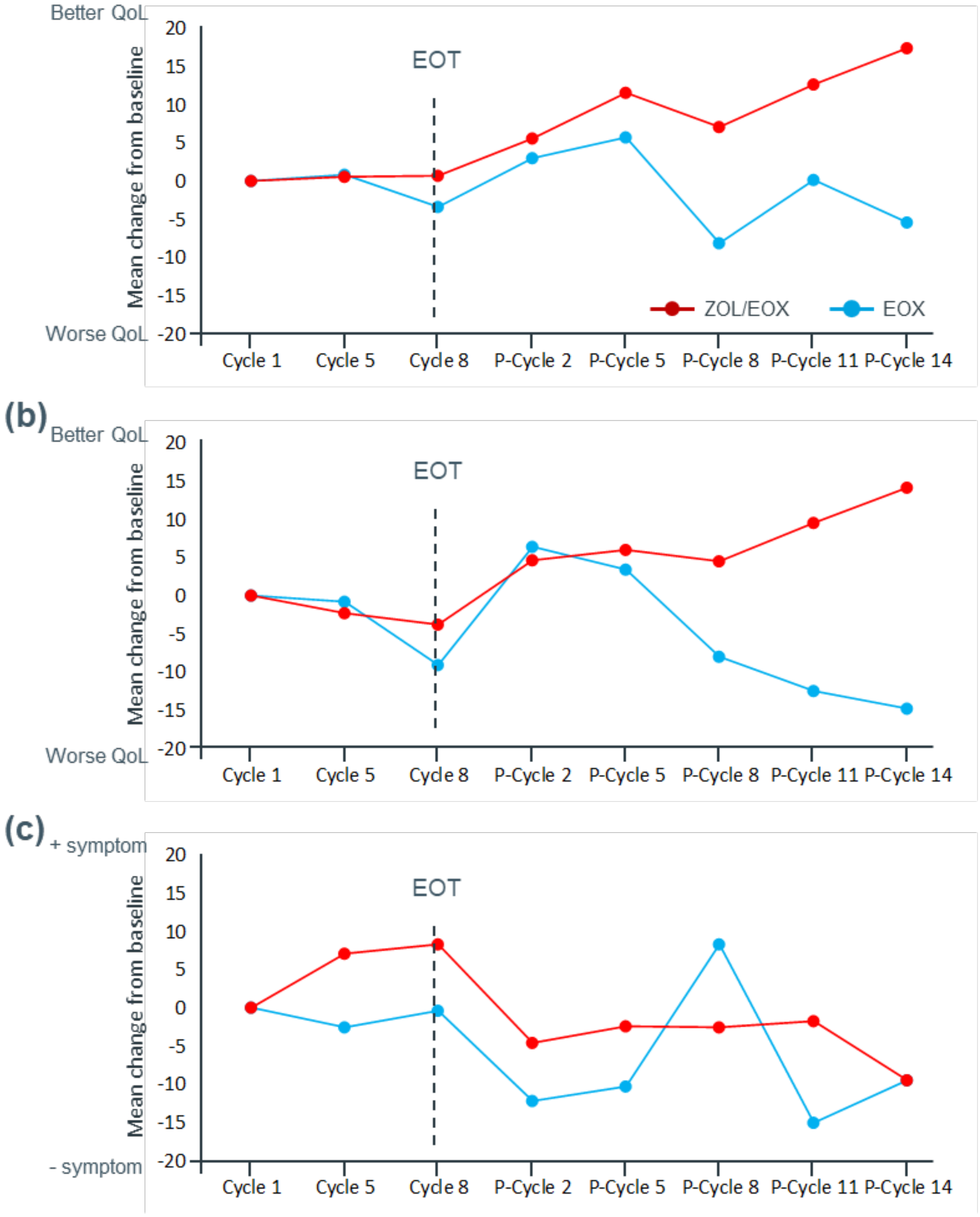

Mean change from baseline throughout the study is provided for (a) global health status, (b) physical functioning, and (c) nausea/vomiting. Abbreviations: EOT: end of EOX treatment; EOX: epirubicin, oxaliplatin, and capecitabine; FAS: full analysis set; P-cycle: post end of EOX treatment cycle; ZOL/EOX: zolbetuximab 800/600 mg/m^2 plus EOX.
